# Gender differences in adult ADHD: Cognitive function assessed by the test of attentional performance

**DOI:** 10.1371/journal.pone.0240810

**Published:** 2020-10-15

**Authors:** Tina Stibbe, Jue Huang, Madlen Paucke, Christine Ulke, Maria Strauss

**Affiliations:** Department of Psychiatry and Psychotherapy, University of Leipzig, Leipzig, Saxony, Germany; Universtiyt of Oviedo (Spain), SPAIN

## Abstract

**Introduction:**

The aim of this study was to assess cognitive differences between male and female adults with Attention Deficit Hyperactivity Disorder (ADHD).

**Methods:**

Patients with an ADHD diagnosis according to the DSM-IV guidelines were included in a cross-sectional study evaluating cognitive measures. 28 women and 41 men from ages 19 to 56 completed self-report questionnaires and performed a computer-based test of attentional performance (TAP). The TAP assesses cognitive functions highly affected in ADHD patients, including working memory, alertness and attention as well as behavioral control and response inhibition.

**Results:**

There were no measurable differences in self-report scales assessing current symptomology between the sexes, however men scored higher on the scale for childhood symptoms. Performance measures for general wakefulness were comparable between men and women, while working memory and behavioral control test results differed. Females reacted significantly slower and more unstable for both the TAP Go/NoGo paradigm and working memory subtest, while also making more errors in the latter.

**Conclusions:**

We found gender-specific effects regarding working memory and behavioral control in this sample of adult patients with ADHD. Further studies are warranted, examining whether these differences relate to differences in clinical presentation and comorbidity patterns between men and women.

## Introduction

Although historically viewed as a childhood mental disorder, Attention Deficit Hyperactivity Disorder (ADHD) is now recognized as a dysfunction affecting a considerable number of adults, with a prevalence of 2–5%, depending on the population [1, 2]. Direct and indirect costs of the disease reach an average of $200 billion each year in the US, about three quarters of which are accounted for by adult patients [3]. Especially when undiagnosed, ADHD can cause several disruptive and antisocial behaviors over the lifespan of patients, ranging from substance abuse and work absenteeism to criminality, further contributing to the socioeconomic burden of the disease [4]. A better understanding of ADHD and the facilitation of its accurate diagnosis, may improve overall disease management and reduce its negative impact.

A distinctive feature of ADHD in children is the high ratio of affected boys vs. girls. Several studies published in the last 20 years showed prevalence differences ranging from 1:1.8 to 1:16 of girls to boys [5–10]. Notably, the gender ratio seemingly narrows to 1:1.6 in female vs. male adults with ADHD [8]. The reasons for these gender differences in various developmental stages of ADHD patients are poorly understood. Some studies suggest that disease persistence into adulthood varies according to gender, with rates around 60% for women [11, 12] and only 35% for men [13]. Other studies suggest a gender difference in the presentation of ADHD, with females more likely to present with inattentive symptoms and males more likely to present with combined symptoms and higher rates of hyperactivity and impulsivity [8, 14]. Impulsive and hyperactive symptoms tend to decrease over time while inattentive symptoms persist, which may lead to the difference in disease prevalence between men and women during childhood and adulthood [14–16].

Along with different symptom presentations, different patterns of comorbidity have also been observed. Males are more likely to experience “externalizing” disorders (e.g. substance or alcohol abuse, antisocial personality disorder, conduct disorder [15, 17–21]), while “internalizing” disorders (e.g. anxiety, depression, somatic symptoms, bulimia [15, 20–22]) are more common in females [22]. These comorbidities can lead to frequent misdiagnoses of adult ADHD patients [23, 24], especially in women [9, 17, 22].

The heterogeneous characteristics between females and males mentioned above raise the question of possible differences in cognitive function. The cognitive areas that are most notably affected in ADHD patients include sustained and focused attention, working memory, inhibitory control and response inhibition [10, 25–28]. Few studies have compared cognitive functionality between men and women. A meta-analysis by Bálint *et al*. in 2009 summarized 25 studies conducted from 1993 to 2007 and concluded that attentional problems of the ADHD groups was directly associated with higher proportions of men in the group [29]. Conversely, some studies have found that females have more attention impairment [30, 31], which leads to the assumption that females more often present with inattentive symptoms [15] and supports the hypothesis that the extent of attentional problems are at least partially associated with the diagnosed ADHD presentation in combination with gender [30]. Interestingly, a study in children and adolescents with ADHD found that poorer performance in executive functions was associated with the less common presentation for each gender. More specifically, boys with the combined/hyperactive-impulsive presentation performed better than girls of the same presentation, and girls with the inattentive presentation showed better test results than boys with inattentive presentation [32]. However, other studies have found no differences in cognitive performance between males and females [18, 33–35]. In summary, results about cognitive differences in male and female adults with ADHD remain inconclusive, while gender-specific distinctions in symptom presentation is widely accepted.

In this explorative study, we aimed to determine whether these observable symptom differences could be measured via standard assessment tools used in our clinic. Our primary objective was to find potential differences between women and men with ADHD in self-report symptom scales and attentional capabilities, assessed by a computer-based attentional performance test. Our secondary objective was to assess correlations between these two measures to find out whether certain symptoms might influence specific areas of cognition.

The long-term goal of these explorations is to use these findings to assist clinicians in better diagnosing adults with ADHD, with special attention to specific gender features, especially for patients with high comorbidities or unclear symptomology.

## Materials and methods

### Subjects

Outpatients with clinically defined ADHD, according to the DSM-IV, were recruited during visits at the ADHD outpatient clinic for adults of the University Hospital Leipzig, Germany. During these visits, experienced psychiatrists and psychologists examined patients’ mental status and obtained their clinical history. Structured clinical interviews and rating scales were applied to confirm the clinical diagnosis of ADHD.

#### Inclusion and exclusion criteria

At screening, ADHD diagnosis was confirmed using both the standardized German version of the ADHD self-rating behavior questionnaire (ADHS-SB, [36]) and the Adult ADHD Self-Report Scale (ASRS-V1.1, [37]). To assess childhood symptoms, the short Wender Utah Rating Scale (WURS-k, [38]) was used. The Beck Depression Inventory (BDI, [39]) was applied to assess the occurrence and severity of comorbid depressive symptoms. Patients were included in the study if they had a confirmed diagnosis of ADHD, a value of 4 or higher in the ASRS and a total score of 30 or higher in the WURS-k. Patients could not be included if they showed signs of acute suicidality or current psychotic symptoms. Also, patients with diagnoses of bipolar disorder, borderline personality disorder, substance or alcohol abuse, schizophrenia and severe major depression were excluded form participation. Pregnancy or lactation was also an exclusion criterion for women.

During the screening procedures, the interviewing clinician also evaluated the intellectual capacities of patients. If, in the opinion of the investigator, patients showed indications of limitations, a short vocabulary test, the German *Wortschatztest* (WST, [40]), was applied to examine whether the intellectual performance would restrict the patient from participating in the study. Only if the WST was below average, a more extensive test for intelligence would have been done and the patient excluded from the study. This process relied on the clinical expertise of the senior physician.

69 patients (28 females, 40.6%) were included into the study. All of the patients identified as cisgender men and cisgender women, respectively. Therefore, we use the terms ‘gender’ and ‘sex’ interchangeably throughout the text. Written informed consent was obtained from all participants prior to any tests and the study was approved by the local ethics committee of the University of Leipzig (EC numbers 199-13-15072013 and 155/15-ff).

### Assessments

For a more thorough assessment of symptomology, the long version of the Conners’ Adult ADHD Rating Scale (CAARS, [41]) was applied in German. The CAARS was chosen as it is a well validated rating scale to assess symptom severity in adults in a German population sample [50, 51]. The 66-item questionnaire gives an in-depth overview of individual core symptom manifestation and severity, which is why it was chosen in this study to follow the shorter questionnaires used at screening, e.g. the ASRS. For the CAARS, standardized T-scores higher than 60 were interpreted as being indicative of ADHD in the CAARS sum and all of its subscales.

Secondly, in order to measure attentional deficits more objectively, the computerized Test of Attentional Performance (TAP, [42]) was used. The TAP is a commonly used validated tool for the evaluation of adults, and is available in Germany. Specifically, the alertness, working memory and Go/NoGo subtests were administered as they can quantify patients’ capacity in cognitive areas highly affected in ADHD patients, such as general wakefulness, information processing and behavioral control [25]. The alertness task took place under two different conditions: uncued (intrinsic alertness) and cued (phasic alertness). The working memory test was used in its medium level of difficulty, as previous studies showed a high frequency of discontinuation when using the high difficulty level due to loss of patient motivation. Lastly, the Go/NoGo subtest was applied with the test form “1 of 2”. The T-scores were corrected for age, education and sex where possible. T-scores of below 40 were interpreted as being indicative of abnormal TAP results and therefore attentional difficulties.

### Statistical analysis

The Pearson chi-square test (χ^2^) was used to investigate gender differences (female vs. male) in educational degree as well as marital and smoker status. For other metrical variables, the independent sample t-Test was used to test for differences between groups based on gender. As the differences for age and other demographical variables between females and males (see Table 1) were not significant, we decided to execute the independent sample t-Test for metrical variables and the Pearson chi-square test (χ^2^) in case of categorical variables to assess potential differences in cognition measured by the CAARS and TAP.

**Table 1:** Basic characteristics and screening scale results for females and males with ADHD.

		Males (n = 41)	Females (n = 28)	Test	df	*p*
**Age [mean (SE)]**		32.6 (1.3)	33.6 (1.8)^a^	T = -0.459	66	0.647
**Age range**		20–56	19–53	-	-	-
**Smoker [n (%)]**	**Yes**	18 (56.3)	14 (43.8)	Χ^2^ = 0.745	1	0.388
	**No**	22 (66.7)	11 (33.3)			
**Marital status [n (%)]**	**Single**	15 (50.0)	15 (50.0)	Χ^2^ = 6.271	5	0.281
	**Married**	6 (75.0)	2 (25.0)			
	**Divorced**	1 (33.3)	2 (66.7)			
	**In a relationship**	6 (60.0)	4 (40.0)			
	**Other**	4 (80.0)	1 (20.0)			
**Educational degree [n (%)]**	**High School**	27 (67.5)	13 (32.5)	Χ^2^ = 4.168	3	0.244
	**Higher Secondary School**	8 (42.1)	11 (57.9)			
	**Lower Secondary School**	5 (62.5)	3 (37.5)			
	**Special Needs School**	1 (100.0)	0 (0.0)			
**Medication**^b^ **[n (%)]**	**None**	25 (55.6)	20 (44.4)	-	-	-
	**Methylphenidate**	3 (75.0)	1 (25.0)	-	-	-
	**Antidepressants**	5 (71.4)	2 (28.6)	-	-	-
	**Neuroleptics**	1 (50.0)	1 (50.0)	-	-	-
	**Antiepileptics**	0 (0.0)	1 (100.0)	-	-	-
	**Thyroxine**	1 (50.0)	1 (50.0)	-	-	-
	**Other**	9 (60.0)	6 (40.0)	-	-	-
	**More than one**	3 (50.0)	3 (50.0)	-	-	-
**WURS-k [mean (SE)]**		43.6 (2.1)	37.4 (2.2)^a^	T = 1.949	66	**0.056**
**ADHS-SB [mean (SE)]**	**Inattention**	18.9 (0.7)	18.0 (0.8)^a^	T = 0.873	66	0.386
	**Hyperactivity**	8.9 (0.6)	9.0 (0.7)^a^	T = -0.168	66	0.867
	**Impulsivity**	6.3 (0.5)	6.9 (0.5)^a^	T = -0.826	66	0.412
**ASRS-V1.1**	**Part A–cut off**	4.6 (0.3)	5.0 (0.2)^a^	T = -1.243	66	0.218
	**Part A–sum**	17.6 (0.5)	17.5 (0.6)	T = 0.164	67	0.870
	**Part B–cut off**	9.1 (0.3)	9.3 (0.3) ^a^	T = -0.400	66	0.690
	**Part B—sum**	32.8 (1.0)	34.5 (0.9)	T = -1.270	67	0.208
**BDI [mean (SE)]**		15.2 (1.7)	14.8 (2.6)^a^	T = 0.123	66	0.902

^a^ Available n = 27

^b^ Medication amounts are depicted as total number and percentage in between genders

In order to investigate the relationship between self-report scales and performance-based measures, correlation analyses were performed. The Spearman rank correlation coefficient was applied to measure strengths in each group. In case of significant correlation, a partial correlation analysis with covariance of gender was conducted to avoid the confounding effect of gender. All statistical analyses were run in SPSS Statistics 24.0 (IBM corp., Armonk, New York). The significance level for all tests was set at p<0.05.

## Results

### Basic and clinical characteristics at screening

In Table 1, main demographic and clinical characteristics of both gender groups are summarized. In line with the overall prevalence of the disorder, fewer females than males were recruited (40.6% vs. 59.4%). However, the groups were well balanced regarding age (total range 19–56, mean±SD = 33.01±8.91) as well as clinical characteristics measured by ADHS-SB, ASRS-v1.1 for ADHD-relevant symptoms and BDI for depressive symptoms. There was a slight tendency toward more medical treatments for men, who received methylphenidate and antidepressants more often than women. The percentage of non-smokers was also higher in the male group vs. the female group. More men also held a high school diploma degree compared to women and were more often married. However, none of these differences reached statistical significance.

No explicit testing for intellectual capacity was performed, however when using educational degree as an indicator, a balanced level of intellect can be assumed. The same proportion of patients, approximately 85% of each group, attended either a higher secondary school or high school (see Table 1).

The only exception in baseline characteristics was the WURS-k scale, with men scoring higher than women. This represents an increased report of occurrence and severity of ADHD symptoms in the male subjects’ childhood compared with the female group. This difference almost reached significance (*p* = 0.056, see Table 1).

### Group differences in self-report measures for symptom severity–CAARS

Consistent with the other ADHD questionnaires assessing current symptoms at screening, there were no significant differences observable between groups in the mean CAARS subscales (*p* ≥ 0.251) (see S1 Table). There were also no differences (*p* ≥ 0.143) between genders for subjects with T scores above 60 in each of the subscales (see Table 2).

**Table 2:** Group differences between males and females scoring above 60 in CAARS subscales.

CAARS subscale	Group	%	X^2^	*p*
**Inattention**	Male (n = 41)	92,68	0.427	0.513
	Female (n = 28)	96,40		
**Hyperactivity**	Male (n = 41)	90,24	0.333	0.564
	Female (n = 28)	85,71		
**Impulsivity**	Male (n = 41)	78,05	0.087	0.768
	Female (n = 28)	75,00		
**Self-Concept**	Male (n = 41)	75,61	0.151	0.698
	Female (n = 28)	71,43		
**DSM-Inattention**	Male (n = 41)	95,12	1.407	0.236
	Female (n = 28)	100,00		
**DSM-Hyperactivity/Impulsivity**	Male (n = 41)	82,93	0.206	0.650
	Female (n = 28)	78,57		
**DSM-Global**	Male (n = 41)	92,68	2.142	0.143
	Female (n = 28)	100,00		
**ADHD-Index**	Male (n = 41)	100,00	-	-
	Female (n = 28)	100,00		

### Group differences in performance-based measures for cognition–TAP

In the alertness subtest of the TAP, general wakefulness is measured, which did not differ between males and females (see Table 3).

**Table 3:** Group differences between males and females in TAP subtests alertness, working memory and Go/No Go paradigm.

TAP subscale	Group	Mean (SE)	df	T	*p*
***Alertness***					
**Intrinsic alertness** ** • Reaction time Median**	Male (n = 41)	42.3 (1.3)	-0.603	67	0.548
	Female (n = 28)	43.7 (2.0)			
**Intrinsic Alertness** ** • SD**	Male (n = 41)	46.0 (1.6)	-0.423	67	0.674
	Female (n = 28)	47.1 (2.4)			
**Phasic Alertness** ** • Reaction time Median**	Male (n = 41)	42.0 (1.1)	0.530	67	0.598
	Female (n = 28)	41.0 (1.5)			
**Phasic Alertness** ** • SD**	Male (n = 41)	45.9 (1.5)	0.014	67	0.989
	Female (n = 28)	45.8 (1.8)			
**Index for phasic alertness**	Male (n = 41)	46.4 (1.4)	0.218	67	0.828
	Female (n = 28)	45.9 (2.0)			
***Working memory***					
**Correct responses**	Male (n = 38)	12.3 (0.4)	0.903	62	0.370
	Female (n = 26)	11.7 (0.5)			
**Errors**	Male (n = 40)	2.2 (0.5)	-2.060	66	**0.043**
	Female (n = 28)	4.3 (1.0)			
**Omissions**	Male (n = 40)	2.7 (0.4)	-1.176	66	0.244
	Female (n = 28)	3.4 (0.5)			
**Reaction time Median (ms)**	Male (n = 40)	650.5 (23.1)	-2.148	65	**0.035**
	Female (n = 27)	736.6 (34.9)			
**SD**	Male (n = 40)	191.4 (12.4)	-2.176	65	**0.033**
	Female (n = 27)	237.1 (17.8)			
***Go/No Go paradigm***					
**Errors**	Male (n = 41)	47.4 (1.2)	-1.201	67	0.234
	Female (n = 28)	49.5 (1.3)			
**Omissions**	Male (n = 41)	48.2 (0.9)	-0.266	67	0.791
	Female (n = 28)	48.5 (0.8)			
**Reaction time Median**	Male (n = 41)	51.5 (1.6)	3.077	55,891	**0.003**
	Female (n = 28)	43.5 (2.1)			
**SD**	Male (n = 41)	47.7 (1.5)	2.552	67	**0.013**
	Female (n = 28)	41.2 (2.2)			

For the alertness and Go/No Go paradigm subscales, mean scores and standard deviations for T scores are depicted. For working memory, raw mean scores and standard deviations were used.

In the TAP subscale assessing working memory, correct responses and measures for omissions did not differ between males and females (*p* = 0.370 and *p* = 0.244, respectively). However, the results for median reaction time (*p* = 0.035) and standard deviation (*p* = 0.033) as well as the number of errors (*p* = 0.043) were significantly higher in females. These results indicate slower and more unstable reactions in the female group, who also made more errors.

In the TAP subscale following the Go/No Go paradigm, behavioral control was tested in both groups. While both groups made comparable errors (*p* = 0.234) and omissions (*p* = 0.791), the median reaction time was significantly slower in women compared with men (*p* = 0.003, see Table 3). At the same time, the standard deviation of the reaction time was significantly higher in women (*p* = 0.013, see Table 3), indicating a more unstable reaction to the given stimuli.

### Correlation analysis between symptom and performance measures

In order to assess whether results of the questionnaire (e.g. occurrence of a predominant symptom in CAARS) were associated with cognitive performance, we used a correlation analysis. There were two significant correlations measurable between self-reports and performance tests (see S2 Table).

The BDI correlated negatively with errors in the Go/No Go subtest of the TAP (ρ = -0.279, *p* = 0.021), suggesting that an increase in depressive symptoms leads to a higher number of errors in the test. After correcting for gender impact, this correlation still showed the approximate same level of significance (ρ = -0.269, *p* = 0.027), suggesting that gender only has a minor or negligible effect. In other words, depressive symptoms worsen the accuracy of the impulse control test Go/No Go, regardless of the patient’s gender.

The subscale for hyperactivity and impulsivity on the CAARS questionnaire had a negative correlation with the standard deviation of the phasic alertness subtest of the TAP (ρ = -0.288, *p* = 0.016). The correlation was not as significant when gender influence was corrected (ρ = -0.244, *p* = 0.045). This suggests that increased hyperactivity and impulsivity leading to a more unstable reaction in phasic alertness might be partly influenced by gender.

## Discussion

Our primary study objective was to examine cognitive differences between men and women in a sample of adult ADHD patients, by 1) using self-report scales like the ADHS-SB [36], ASRS [37], WURS-k [38] and CAARS [41] to assess symptom severity and characteristics, and 2) using a computer-based performance test (TAP, [42]) to measure attentional features affected in ADHD such as alertness, working memory or behavioral control.

Interestingly, in the first part using scales that rely on the self-assessment of current symptomology, namely the ASRS, ADHS-SB and CAARS, did not differentiate between women and men. To this date, there is no consensus regarding gender differences in symptom severity in adults, as some studies have concluded that no differences exist [18, 33, 43–45], while other groups reported different results for men and women, especially in the subscales for inattention and hyperactivity/impulsivity [30, 46–49].

The only scale that differed between men and women in our results was the WURS-k, focusing on reports about symptoms during patients’ childhood, with men scoring significantly higher than women. These results are in line with a previous study comparing male and female ADHD patients [50] and could be explained by both the different nature of symptoms and also the difference in perception and reporting of childhood symptoms between genders [51]. In general, females with ADHD have inattentive, depression- and anxiety-like symptoms and are therefore often misdiagnosed as a child [17, 22, 44, 49, 50, 52, 53]. These findings are corroborated by a study using the WURS-k as a self-report measure, where females scored higher on items regarding sadness and emotional distress [51]. Symptoms in boys on the other hand are primarily characterized by hyperactivity and impulsivity, which may decrease over time and result in a more subtle manifestation in adulthood, comparable to that of females [15, 54, 55].

In the second part, we objectified core symptoms such as inattention, impulsivity and hyperactivity by measuring performance on the computerized subtests working memory, alertness and the Go/No Go of the TAP [42]. In our test sample, gender did not dictate different results for general wakefulness measured by the alertness subtest. Few studies have looked specifically at gender differences in adult ADHD patients when performing attention tasks, and more often compare ADHD patients to healthy controls or other mental disorders [15]. The available data on attention tests similar to the TAP test, e.g. the Stroop Word Color Test [56] or the Continuous Performance Test (CPT, [57]), is inconclusive. A meta-analysis by Bálint and colleagues in 2009 found an association of the percentage of men in the test sample with poorer performance in the Stroop test for attention [29]. One study, that was not included in this review, however saw no differences in the Stroop Color and Word test [58], which was also shown in an earlier publication of the group [59]. A more recent publication of Edebol *et al*. from 2013 observed a significant impairment of women in measures for inattention in a study comparing 55 ADHD patients [31].

On the other hand, we saw significant differences in working memory and impulse control results. Female patients had significantly lower measures for reaction time in the working memory subtest, with a less stable reaction. Moreover, women made more errors than men. These results are in contrast to early studies from Katz *et al*. published in 1998, which suggested a better score for women than men [60] measured by scales such as the Wechsler Memory Scale and Symbol Digit tests [61, 62]. A more recent study of Schweitzer *et al*. however was confirmed by our findings, as the group also saw significantly better measures of working memory for men over women. Notably, the group also analyzed whether presentations could explain cognitive performance differences, but found no such association between ADHD presentation and working memory deficits [63]. With a different approach, the group of Valera *et al*. examined the neural activation of adult ADHD patients by performing functional MRI (fMRI) scans while the patient performed working memory tasks. The results indicated that compared to same sex controls, ADHD males had less neural activation than females. Moreover, they found negative correlations between hyperactive symptoms in men and inattentive symptoms in women, suggesting a difference in presentation between the sexes [64].

The second TAP subtest that yielded differences between sexes in our study, was the Go/No Go subtest with women again reacting significantly slower and less stable than men. Deficits in inhibitory control are deemed a core symptom of ADHD, so much so that it has been studied as a differentiating diagnostic marker between Autism spectrum disorders and ADHD [65]. Other groups consider it a cognitive endophenotype in conclusion of studies examining the genetic risk of ADHD in association with inhibitory control ability of parents and their children [66, 67]. Interestingly, in 2009, the Goos group found a difference in effects when looking at the influence of maternal vs. paternal inhibition abilities. According to their report, inhibitory control of fathers were more influential on children’s behavior control abilities than that of their mothers, suggesting at least a partial effect of gender down the line [67]. The data on gender differences in adults is again scarce, however a study using fMRI analysis of ADHD adults performing a Go/No Go task from 2014 reported no differences between genders [68]. On the other hand, the Wright group showed that omission errors were more common in males with ADHD during the Go/No Go task, indicating higher rates of inattention [69]. Interestingly, a study of healthy adults done by Liu *et al*. observed faster reactions in males than females in a Go/No Go task, while accuracy was similar for both groups, which is in accordance with our results [70].

We can only speculate as to why women in our study population did not perform as well in these TAP subtests. One explanation could be that women with ADHD may have problems with maintaining concentration over extended periods of time, which may be supported by the fact that the inattentive presentation is more common among women. The TAP tests were completed in order of increasing complexity: alertness; Go/NoGo; working memory. Particularly in a stress-inducing situation like a computer performance test, this might have resulted in worse performance for women in the latter, more complex tasks. Some preclinical studies also linked stress stimuli to hyperarousal in females, which could have led to wrong actions and less stable performance in the TAP tests [71]. Another important point in this context is the potential gender bias in the questionnaires. Women more often present with inattentive symptoms; therefore, when they achieve total scores as high as predominantly hyperactive, impulsive males on these questionnaires, it would indicate higher impairment in the attention-associated factors [9]. As shown in studies with children, these subjective measures have different gender effects than objective measures, leading to a worse performance in the latter [72]. In conclusion, women might seem equally impaired according to the total questionnaire scores, however show an elevated level of impairment in objective performance tests.

Lastly, we performed correlation analyses of the performance-based and self-report scales. In a first step we did Spearman rank correlation analyses for all tests. In case of a significant result, we corrected these correlations for gender effects in a second step to see whether the result would change. Only two correlations between self-report and performance tests were significant. While the higher scores on the BDI led to more errors in the Go/No Go subtest, the CAARS Hyperactivity/Impulsivity subscores correlated with decreased stability of the phasic alertness subtest. Only the latter seemed to be slightly influenced by gender, while impulse control was solely dependent on the severity of depressive symptoms. Taken together, it seems self-reported symptoms such as hyperactivity and inattention did not automatically worsen performance in either women or men, with the exception of the CAARS hyperactivity/impulsivity subscale indicating a less stable wakefulness on a given cue and the BDI leading to more errors when assessing impulse control. These correlations were only partly influenced by gender and rather suggest a connection to the presentation of ADHD. As a previous study in children suggested, the predominant symptom is more indicative of cognitive performance than the gender itself [73]. Another study with children concluded that performance in cognition worsened whenever the presentation was less common among the respective gender, i.e. inattentive boys scored lower on cognitive tests than boys with the combined presentation and vice versa for girls [32]. On the other hand, a study by Song *et al*. reported similar executive deficits seen in children with the inattentive and combined presentation [74]. To our knowledge, there are no studies in adults with ADHD analyzing the correlation between symptom presentation and attentional performance tests.

Limitations of this study include the lack of control of the possible influence of pharmacotherapy and psychotherapy on the cognitive performance of ADHD patients and the unbalanced numbers of men vs. women in an overall small sample size, making it impossible to generalize our findings. The measures used were also reliant on a subject`s self-assessment. Moreover, we did not differentiate between presentations and a control group of healthy subjects was not included in the analysis to compare gender effects in the general population.

In summary, our results indicate that women and men with ADHD may differ in cognitive capacities, with women being more impaired than men in working memory and impulse control. Whether these effects are comparable to gender differences and hormonal influence in the general public, as shown by other studies [30, 75], or whether they are a consequence of the mental disorder itself or a delayed onset of treatment due to mis- or underdiagnosis, remains unclear and must be studied in further detail.

There is, however, certainty that the cognitive performance of ADHD patients varies depending on comorbidities and presentation characteristics [15, 50], which in turn has been linked to gender differences. We therefore agree with the general consensus that individual cognitive features should be thoroughly assessed using neurocognitive tests in clinical practice [25, 76, 77]. Although the current diagnostic criteria do not take gender differences into account, their clinical presentation does verifiably differ. Moreover, men and women might benefit from other combinations of treatments based on comorbidities, predominant symptoms and therewith cognitive difficulties.

## Supporting information

S1 Table Group differences between males and females in CAARS subscales. (XLS)

S2 Table Correlations of the subtests of the TAP with self-report scales WURS-k, BDI and CAARS. (XLS)

## References

[1] Asherson P, Buitelaar J, Faraone SV, Rohde LA (2016) Adult attention-deficit hyperactivity disorder: key conceptual issues. The Lancet Psychiatry 3 (6): 568–578. 10.1016/S2215-0366(16)30032-3 27183901

[2] Fayyad J, Sampson NA, Hwang I, Adamowski T, Aguilar-Gaxiola S et al (2017) The descriptive epidemiology of DSM-IV Adult ADHD in the World Health Organization World Mental Health Surveys. Attention deficit and hyperactivity disorders 9 (1): 47–65. 10.1007/s12402-016-0208-3 27866355 PMC5325787

[3] Barkley RA (2020) The High Economic Costs Associated with ADHD. The ADHD Report 28 (3): 10–12. Available: https://www.awmf.org/uploads/tx_szleitlinien/028-045k_S3_ADHS_2018-06.pdf.

[4] Franke B, Michelini G, Asherson P, Banaschewski T, Bilbow A et al (2018) Live fast, die young? A review on the developmental trajectories of ADHD across the lifespan. European neuropsychopharmacology: the journal of the European College of Neuropsychopharmacology 28 (10): 1059–1088. 30195575 10.1016/j.euroneuro.2018.08.001 PMC6379245

[5] Nøvik TS, Hervas A, Ralston SJ, Dalsgaard S, Rodrigues Pereira R et al (2006) Influence of gender on attention-deficit/hyperactivity disorder in Europe—ADORE. European child & adolescent psychiatry 15 Suppl 1: I15–24. 17177011 10.1007/s00787-006-1003-z

[6] Arnett AB, Pennington BF, Willcutt EG, DeFries JC, Olson RK (2015) Sex differences in ADHD symptom severity. Journal of child psychology and psychiatry, and allied disciplines 56 (6): 632–639. 10.1111/jcpp.12337 25283790 PMC4385512

[7] Biederman J, Mick E, Faraone SV, Braaten E, Doyle A et al (2002) Influence of gender on attention deficit hyperactivity disorder in children referred to a psychiatric clinic. The American journal of psychiatry 159 (1): 36–42. 10.1176/appi.ajp.159.1.36 11772687

[8] Willcutt EG (2012) The prevalence of DSM-IV attention-deficit/hyperactivity disorder. A meta-analytic review. Neurotherapeutics: the journal of the American Society for Experimental NeuroTherapeutics 9 (3): 490–499. 22976615 10.1007/s13311-012-0135-8 PMC3441936

[9] Mowlem FD, Rosenqvist MA, Martin J, Lichtenstein P, Asherson P et al (2019) Sex differences in predicting ADHD clinical diagnosis and pharmacological treatment. European child & adolescent psychiatry 28 (4): 481–489. 30097723 10.1007/s00787-018-1211-3 PMC6445815

[10] Magnin E, Maurs C (2017) Attention-deficit/hyperactivity disorder during adulthood. Revue neurologique 173 (7–8): 506–515. 10.1016/j.neurol.2017.07.008 28844700

[11] Biederman J, Petty CR, Monuteaux MC, Fried R, Byrne D et al (2010) Adult psychiatric outcomes of girls with attention deficit hyperactivity disorder. 11-year follow-up in a longitudinal case-control study. The American journal of psychiatry 167 (4): 409–417. 10.1176/appi.ajp.2009.09050736 20080984

[12] Hinshaw SP, Owens EB, Zalecki C, Huggins SP, Montenegro-Nevado AJ et al (2012) Prospective follow-up of girls with attention-deficit/hyperactivity disorder into early adulthood. Continuing impairment includes elevated risk for suicide attempts and self-injury. Journal of consulting and clinical psychology 80 (6): 1041–1051. 10.1037/a0029451 22889337 PMC3543865

[13] Biederman J, Petty CR, Evans M, Small J, Faraone SV (2010) How persistent is ADHD? A controlled 10-year follow-up study of boys with ADHD. Psychiatry Research 177 (3): 299–304. 10.1016/j.psychres.2009.12.010 20452063 PMC2881837

[14] Li T, Mota NR, Galesloot TE, Bralten J, Buitelaar JK et al (2019) ADHD symptoms in the adult general population are associated with factors linked to ADHD in adult patients. European neuropsychopharmacology: the journal of the European College of Neuropsychopharmacology 29 (10): 1117–1126. 31378654 10.1016/j.euroneuro.2019.07.136

[15] Williamson D, Johnston C (2015) Gender differences in adults with attention-deficit/hyperactivity disorder. A narrative review. Clinical psychology review 40: 15–27. Accessed 9 May 2018. 10.1016/j.cpr.2015.05.005 26046624

[16] Francx W, Zwiers MP, Mennes M, Oosterlaan J, Heslenfeld D et al (2015) White matter microstructure and developmental improvement of hyperactive/impulsive symptoms in attention-deficit/hyperactivity disorder. Journal of child psychology and psychiatry, and allied disciplines 56 (12): 1289–1297. 10.1111/jcpp.12379 25581343 PMC4499023

[17] Nussbaum NL (2012) ADHD and female specific concerns: a review of the literature and clinical implications. Journal of attention disorders 16 (2): 87–100. 10.1177/1087054711416909 21976033

[18] Biederman J, Faraone SV, Monuteaux MC, Bober M, Cadogen E (2004) Gender effects on attention-deficit/hyperactivity disorder in adults, revisited. Biological psychiatry 55 (7): 692–700. Accessed 9 May 2018. 10.1016/j.biopsych.2003.12.003 15038997

[19] Rucklidge JJ (2010) Gender differences in attention-deficit/hyperactivity disorder. The Psychiatric clinics of North America 33 (2): 357–373. Accessed 9 May 2018. 10.1016/j.psc.2010.01.006 20385342

[20] Cortese S, Faraone SV, Bernardi S, Wang S, Blanco C (2016) Gender differences in adult attention-deficit/hyperactivity disorder. Results from the National Epidemiologic Survey on Alcohol and Related Conditions (NESARC). The Journal of clinical psychiatry 77 (4): e421–8. 10.4088/JCP.14m09630 27137426

[21] Solberg BS, Halmøy A, Engeland A, Igland J, Haavik J et al (2018) Gender differences in psychiatric comorbidity: a population-based study of 40 000 adults with attention deficit hyperactivity disorder. Acta psychiatrica Scandinavica 137 (3): 176–186. 10.1111/acps.12845 29266167 PMC5838558

[22] Quinn PO (2008) Attention-deficit/hyperactivity disorder and its comorbidities in women and girls. An evolving picture. Current psychiatry reports 10 (5): 419–423. 10.1007/s11920-008-0067-5 18803916

[23] Ginsberg Y, Quintero J, Anand E, Casillas M, Upadhyaya HP (2014) Underdiagnosis of attention-deficit/hyperactivity disorder in adult patients. A review of the literature. The primary care companion for CNS disorders 16 (3). 10.4088/PCC.13r01600 PMC4195639 25317367

[24] Barkley RA, Brown TE (2008) Unrecognized Attention-Deficit/Hyperactivity Disorder in Adults Presenting with Other Psychiatric Disorders. CNS Spectrums 13 (11): 977–984. Available: https://www.cambridge.org/core/services/aop-cambridge-core/content/view/81DB99B6CD7870AE879CD03D4C1067C3/S1092852900014036a.pdf/div-class-title-unrecognized-attention-deficit-hyperactivity-disorder-in-adults-presenting-with-other-psychiatric-disorders-div.pdf. 10.1017/s1092852900014036 19037178

[25] Fuermaier ABM, Fricke JA, Vries SM de, Tucha L, Tucha O (2019) Neuropsychological assessment of adults with ADHD: A Delphi consensus study. Applied neuropsychology. Adult 26 (4): 340–354. 10.1080/23279095.2018.1429441 29424567

[26] Pievsky MA, McGrath RE (2018) The Neurocognitive Profile of Attention-Deficit/Hyperactivity Disorder: A Review of Meta-Analyses. Archives of clinical neuropsychology: the official journal of the National Academy of Neuropsychologists 33 (2): 143–157. 29106438 10.1093/arclin/acx055

[27] Rubia K (2018) Cognitive Neuroscience of Attention Deficit Hyperactivity Disorder (ADHD) and Its Clinical Translation. Frontiers in Human Neuroscience 12 10.3389/fnhum.2018.00012 29651240 PMC5884954

[28] LeRoy A, Jacova C, Young C (2019) Neuropsychological Performance Patterns of Adult ADHD Subtypes. Journal of attention disorders 23 (10): 1136–1147. 10.1177/1087054718773927 29771179

[29] Bálint S, Czobor P, Komlósi S, Mészáros A, Simon V et al (2009) Attention deficit hyperactivity disorder (ADHD). Gender- and age-related differences in neurocognition. Psychological medicine 39 (8): 1337–1345. 10.1017/S0033291708004236 18713489

[30] Retz-Junginger P, Rösler M, Jacob C, Alm B, Retz W (2010) Gender differences in self- and investigator-rated psychopathology in adult attention-deficit/hyperactivity disorder. Attention deficit and hyperactivity disorders 2 (2): 93–101. Accessed 9 5 2018 10.1007/s12402-010-0024-0 21432594

[31] Edebol H, Helldin L, Norlander T (2013) Measuring adult Attention Deficit Hyperactivity Disorder using the Quantified Behavior Test Plus. PsyCh journal 2 (1): 48–62. 10.1002/pchj.17 24294490 PMC3832237

[32] Wodka EL, Mostofsky SH, Prahme C, Gidley Larson JC, Loftis C et al (2008) Process examination of executive function in ADHD. Sex and subtype effects. The Clinical neuropsychologist 22 (5): 826–841. 10.1080/13854040701563583 18609314 PMC2575661

[33] Biederman J, Kwon A, Aleardi M, Chouinard V-A, Marino T et al (2005) Absence of gender effects on attention deficit hyperactivity disorder: findings in nonreferred subjects. The American journal of psychiatry 162 (6): 1083–1089. Accessed 21 December 2018. 10.1176/appi.ajp.162.6.1083 15930056

[34] Arcia E, Conners CK (1998) Gender differences in ADHD. Journal of developmental and behavioral pediatrics: JDBP 19 (2): 77–83. 10.1097/00004703-199804000-00003 9584935

[35] Silva KL, Guimarães-da-Silva PO, Grevet EH, Victor MM, Salgado CAI et al (2013) Cognitive deficits in adults with ADHD go beyond comorbidity effects. Journal of attention disorders 17 (6): 483–488. Accessed 9 May 2018. 10.1177/1087054711434155 22344317

[36] Rösler M, Retz W, Retz-Junginger P, Thome J, Supprian T et al (2004) Instrumente zur Diagnostik der Aufmerksamkeitsdefizit-/Hyperaktivitätsstörung (ADHS) im ErwachsenenalterSelbstbeurteilungsskala (ADHS-SB) und Diagnosecheckliste (ADHS-DC). Der Nervenarzt 75 (9): 888–895. 10.1007/s00115-003-1622-2 15378249

[37] Kessler RC, Adler L, Ames M, Demler O, Faraone S et al (2005) The World Health Organization Adult ADHD Self-Report Scale (ASRS): a short screening scale for use in the general population. Psychological medicine 35 (2): 245–256. 10.1017/s0033291704002892 15841682

[38] Retz-Junginger P, Retz W, Blocher D, Weijers HG, Trott GE et al (2002) Wender Utah Rating Scale (WURS-k) Die deutsche Kurzform zur retrospektiven Erfassung des hyperkinetischen Syndroms bei Erwachsenen. Der Nervenarzt 73 (9): 830–838. 10.1007/s00115-001-1215-x 12215873

[39] Beck AT, Hautzinger M, Steer RA (1995) Beck-Depressions-Inventar (BDI). Bern: Huber. 2 Einheiten p.

[40] Schmidt K-H, Metzler P (1994) Wortschatztest. WST. Diagnostica 3 (40): 293–297.

[41] Christiansen H, Hirsch O, Abdel-Hamid M et al. (2014) Conners Skalen zu Aufmerksamkeit und Verhalten für Erwachsene (CAARS). Deutschsprachige Adaptation der Conners' Adult ADHD Rating Scale (CAARS) von C. Keith Conners, Drew Erhard und Elizabeth Sparrow. Hans Huber, Bern.

[42] Zimmermann P, Fimm B (2012) TAP Testbatterie zur Aufmerksamkeitsprüfung. Version 2.3. Herzogenrath: Psytest.

[43] Corbisiero S, Hartmann-Schorro RM, Riecher-Rössler A, Stieglitz R-D (2017) Screening for Adult Attention-Deficit/Hyperactivity Disorder in a Psychiatric Outpatient Population with Specific Focus on Sex Differences. Frontiers in psychiatry 8: 115 Accessed 21 December 2018. 10.3389/fpsyt.2017.00115 28713294 PMC5491936

[44] Leung VM, Chan LF (2017) A Cross-sectional Cohort Study of Prevalence, Co-Morbidities, and Correlates of Attention-deficit Hyperactivity Disorder among Adult Patients Admitted to the Li Ka Shing Psychiatric Outpatient Clinic, Hong Kong. East Asian archives of psychiatry: official journal of the Hong Kong College of Psychiatrists = Dong Ya jing shen ke xue zhi: Xianggang jing shen ke yi xue yuan qi kan 27 (2): 63–70. 28652499

[45] DuPaul GJ, Schaughency EA, Weyandt LL, Tripp G, Kiesner J et al (2001) Self-report of ADHD symptoms in university students: cross-gender and cross-national prevalence. Journal of learning disabilities 34 (4): 370–379. 10.1177/002221940103400412 15503581

[46] Morin AJS, Tran A, Caci H (2016) Factorial Validity of the ADHD Adult Symptom Rating Scale in a French Community Sample: Results From the ChiP-ARD Study. Journal of attention disorders 20 (6): 530–541. Accessed 21 December 2018. 10.1177/1087054713488825 23729493

[47] Christiansen H, Kis B, Hirsch O, Philipsen A, Henneck M et al (2011) German validation of the Conners Adult ADHD Rating Scales-self-report (CAARS-S) I: factor structure and normative data. European psychiatry: the journal of the Association of European Psychiatrists 26 (2): 100–107. Accessed 21 December 2018. 20619613 10.1016/j.eurpsy.2009.12.024

[48] Adler LA, Faraone SV, Sarocco P, Atkins N, Khachatryan A (2019) Establishing US norms for the Adult ADHD Self-Report Scale (ASRS-v1.1) and characterising symptom burden among adults with self-reported ADHD. International journal of clinical practice 73 (1): e13260 10.1111/ijcp.13260 30239073 PMC6585602

[49] Vildalen VU, Brevik EJ, Haavik J, Lundervold AJ (2016) Females With ADHD Report More Severe Symptoms Than Males on the Adult ADHD Self-Report Scale. Journal of attention disorders. Accessed 21 December 2018. 10.1177/1087054716659362 27461728

[50] Das D, Vélez JI, Acosta MT, Muenke M, Arcos-Burgos M et al (2016) Retrospective assessment of childhood ADHD symptoms for diagnosis in adults: validity of a short 8-item version of the Wender-Utah Rating Scale. Attention deficit and hyperactivity disorders 8 (4): 215–223. Accessed 20 December 2018. 10.1007/s12402-016-0202-9 27510231

[51] Retz-Junginger P, Retz W, Schneider M, Schwitzgebel P, Steinbach E et al (2007) Der Einfluss des Geschlechts auf die Selbstbeschreibung kindlicher ADHS-Symptome. Der Nervenarzt 78 (9): 1046–1051. 10.1007/s00115-006-2242-4 17268790

[52] Kolla NJ, van der Maas M, Toplak ME, Erickson PG, Mann RE et al (2016) Adult attention deficit hyperactivity disorder symptom profiles and concurrent problems with alcohol and cannabis: sex differences in a representative, population survey. BMC psychiatry 16: 50 Accessed 21 December 2018. 10.1186/s12888-016-0746-4 26920911 PMC4769555

[53] Gershon J (2002) A meta-analytic review of gender differences in ADHD. Journal of attention disorders 5 (3): 143–154. 10.1177/108705470200500302 11911007

[54] McLean A, Dowson J, Toone B, Young S, Bazanis E et al (2004) Characteristic neurocognitive profile associated with adult attention-deficit/hyperactivity disorder. Psychological medicine 34 (4): 681–692. 10.1017/S0033291703001296 15099422

[55] Biederman J, Mick E, Faraone SV (2000) Age-dependent decline of symptoms of attention deficit hyperactivity disorder: impact of remission definition and symptom type. The American journal of psychiatry 157 (5): 816–818. Accessed 21 December 2018. 10.1176/appi.ajp.157.5.816 10784477

[56] Stroop JR (1935) Studies of interference in serial verbal reactions. Journal of Experimental Psychology 18 (6): 643–662.

[57] Rosvold HE, Mirsky AF, Sarason I, Bransome ED JR., Beck LH (1956) A continuous performance test of brain damage. Journal of Consulting Psychology 20 (5): 343–350. 10.1037/h0043220 13367264

[58] Robison RJ, Reimherr FW, Marchant BK, Faraone SV, Adler LA et al (2008) Gender differences in 2 clinical trials of adults with attention-deficit/hyperactivity disorder. A retrospective data analysis. The Journal of clinical psychiatry 69 (2): 213–221. 10.4088/jcp.v69n0207 18211131

[59] Faraone SV, Biederman J, Spencer T, Michelson D, Adler L et al (2005) Atomoxetine and stroop task performance in adult attention-deficit/hyperactivity disorder. Journal of child and adolescent psychopharmacology 15 (4): 664–670. 10.1089/cap.2005.15.664 16190797

[60] Katz LJ, Goldstein G, Geckle M (1998) Neuropsychological and personality differences between men and women with ADHD. J Atten Disord 2 (4): 239–247.

[61] Wechsler D (1987) Wechsler Memory Scale-Revised. San Antonio, Tx: The Psychological Corporation.

[62] Wechsler D (1981) Wechsler Adult Intelligence-Revised. San Antonio, Tx: The Psychological Corporation.

[63] Schweitzer JB, Hanford RB, Medoff DR (2006) Working memory deficits in adults with ADHD: is there evidence for subtype differences. Behavioral and brain functions: BBF 2: 43 10.1186/1744-9081-2-43 17173676 PMC1762010

[64] Valera EM, Brown A, Biederman J, Faraone SV, Makris N et al (2010) Sex differences in the functional neuroanatomy of working memory in adults with ADHD. The American journal of psychiatry 167 (1): 86–94. 10.1176/appi.ajp.2009.09020249 19884224 PMC3777217

[65] Ishii-Takahashi A, Takizawa R, Nishimura Y, Kawakubo Y, Kuwabara H et al (2014) Prefrontal activation during inhibitory control measured by near-infrared spectroscopy for differentiating between autism spectrum disorders and attention deficit hyperactivity disorder in adults. NeuroImage. Clinical 4: 53–63. 10.1016/j.nicl.2013.10.002 24298446 PMC3842411

[66] Slaats-Willemse D, Swaab-Barneveld H, Sonneville L de, van der Meulen E, Buitelaar J (2003) Deficient response inhibition as a cognitive endophenotype of ADHD. Journal of the American Academy of Child and Adolescent Psychiatry 42 (10): 1242–1248. 10.1097/00004583-200310000-00016 14560175

[67] Goos LM, Crosbie J, Payne S, Schachar R (2009) Validation and extension of the endophenotype model in ADHD patterns of inheritance in a family study of inhibitory control. The American journal of psychiatry 166 (6): 711–717. 10.1176/appi.ajp.2009.08040621 19448185

[68] Morein-Zamir S, Dodds C, van Hartevelt TJ, Schwarzkopf W, Sahakian B et al (2014) Hypoactivation in right inferior frontal cortex is specifically associated with motor response inhibition in adult ADHD. Human brain mapping 35 (10): 5141–5152. 10.1002/hbm.22539 24819224 PMC4336557

[69] Wright L, Lipszyc J, Dupuis A, Thayapararajah SW, Schachar R (2014) Response inhibition and psychopathology: a meta-analysis of go/no-go task performance. Journal of abnormal psychology 123 (2): 429–439. 10.1037/a0036295 24731074

[70] Liu J, Zubieta J-K, Heitzeg M (2012) Sex differences in anterior cingulate cortex activation during impulse inhibition and behavioral correlates. Psychiatry Research 201 (1): 54–62. 10.1016/j.pscychresns.2011.05.008 22285718 PMC3289751

[71] Bangasser DA, Wicks B (2017) Sex-specific mechanisms for responding to stress. Journal of neuroscience research 95 (1–2): 75–82. 10.1002/jnr.23812 27870416 PMC5120612

[72] Slobodin O, Davidovitch M (2019) Gender Differences in Objective and Subjective Measures of ADHD Among Clinic-Referred Children. Frontiers in Human Neuroscience 13 10.3389/fnhum.2019.00013 31920599 PMC6923191

[73] Ahmadi N, Mohammadi MR, Araghi SM, Zarafshan H (2014) Neurocognitive Profile of Children with Attention Deficit Hyperactivity Disorders (ADHD): A comparison between subtypes. Iranian Journal of Psychiatry 9 (4): 197–202. 25792987 PMC4361821

[74] Song Y (2015) Cognitive Function in Attention Deficit Hyperactivity Disorder. In: Norvilitis JM, editor. ADHD—New Directions in Diagnosis and Treatment: InTech.

[75] Pletzer B, Harris T-A, Ortner T (2017) Sex and menstrual cycle influences on three aspects of attention. Physiology & behavior 179: 384–390. Available: https://www.rivistadipsichiatria.it/r.php?v=3142&a=31249&l=337008&f=allegati/03142_2019_02/fulltext/05-Salvi%20(84-89).pdf. Accessed 2 January 2019. 28694156 10.1016/j.physbeh.2017.07.012 PMC7115981

[76] Nikolas MA, Marshall P, Hoelzle JB (2019) The role of neurocognitive tests in the assessment of adult attention-deficit/hyperactivity disorder. Psychological assessment 31 (5): 685–698. 10.1037/pas0000688 30730189

[77] Tourjman SV, Potvin S, Corbalan F, Djouini A, Purdon SE et al (2019) Rapid screening for cognitive deficits in attention deficit and hyperactivity disorders with the screen for cognitive impairment in psychiatry. Attention deficit and hyperactivity disorders 11 (2). Available: https://pubmed.ncbi.nlm.nih.gov/30225804/. 10.1007/s12402-018-0268-7 30225804

